# A Handbook of Hygiene

**Published:** 1895-12

**Authors:** 


*A Handbook of Hygiene.* By A. M. DAVIES. Pp. xii., 590. London: CHARLES GRIFFIN AND COMPANY, LIMITED. 1895.

Dr. Davies, the late Assistant Professor of Hygiene in the Army Medical School at Netley, makes no claim for originality in this volume. He has aimed at collecting together in small compass, with the selective aid of his own experience, all the most valuable information contained in the larger, and to many students inaccessible, text-books. The selection is on the whole well done, and the book contains a very large amount of useful information.

As a handy and trustworthy reference book for medical officers serving abroad, it will be useful; but omissions, necessitated by the scheme of the work, stand in the way of its serving as a complete text-book for the study of public health.

